# Quantification of left atrial strain and strain rate using cardiovascular magnetic resonance myocardial feature tracking

**DOI:** 10.1186/1532-429X-17-S1-P66

**Published:** 2015-02-03

**Authors:** Johannes T Kowallick, Shelby Kutty, Frank Edelmann, Amedeo Chiribiri, Adriana Villa, Michael Steinmetz, Jan M Sohns, Wieland Staab, Nuno Bettencourt, Christina Unterberg-Buchwald, Gerd Hasenfuss, Joachim Lotz, Andreas Schuster

**Affiliations:** 1 Division of Imaging Sciences and Biomedical Engineering, The Rayne Institute, St. Thomas' Hospital, King's College London, London, UK; 2 Institute for Diagnostic and Interventional Radiology, University Medical Centre Göttingen, Georg-August-University Göttingen, Göttingen, Germany; 3 DZHK (German Centre for Cardiovascular Research), DZHK Partner Site Göttingen, Göttingen, Germany; 4 Children's Hospital and Medical Center Joint Division of Pediatric Cardiology, University of Nebraska / Creighton University, Omaha, NE, USA; 5 Department of Cardiology and Pneumology, University Medical Centre Göttingen, Georg-August-University, Göttingen, Germany; 6 Clinic for Pediatric Cardiology and Intensive Care Medicine, University Medical Centre Göttingen, Georg-August-University, Göttingen, Germany; 7 Cardiology Department, Centro Hospitalar de Gaia/Espinho, Vila Nova de Gaia, Portugal

## Background

Cardiovascular Magnetic Resonance myocardial feature tracking (CMR-FT) is a quantitative technique tracking tissue voxel motion on standard steady-state free precession (SSFP) cine images to assess ventricular myocardial deformation. The importance of left atrial (LA) deformation assessment is increasingly recognized and can be assessed with echocardiographic speckle tracking. However atrial deformation quantification has never previously been demonstrated with CMR. We sought to determine the feasibility and reproducibility of CMR-FT for quantitative LA strain and strain rate (SR) analysis.

## Methods

10 healthy volunteers, 10 patients with hypertrophic cardiomyopathy (HCM) and 10 patients with heart failure and preserved ejection fraction (HFpEF) were studied at 1.5 Tesla. LA longitudinal strain and SR parameters were derived from SSFP cine images using dedicated CMR-FT software (2D CPA MR, TomTec, Germany). LA performance was analyzed using 4- and 2-chamber views including LA reservoir function (total strain [e_s_], peak positive SR [SR_s_]), LA conduit function (passive strain [e_e_], peak early negative SR [SR_e_]) and LA booster pump function (active strain [e_a_], late peak negative SR [SR_a_]).

## Results

In all subjects LA strain and SR parameters could be derived from SSFP images. There was impaired LA reservoir function in HCM and HFpEF (e_s_ [%]: HCM 22.1±5.5, HFpEF 16.3±5.8, Controls 29.1±5.3, p<0.01; SR_s_ [s^-1^]: HCM 0.9±0.2, HFpEF 0.8±0.3, Controls 1.1±0.2, p<0.05) and impaired LA conduit function as compared to healthy controls (e_e_ [%]: HCM 10.4±3.9, HFpEF 11.9±4.0, Controls 21.3±5.1, p<0.001; SR_e_ [s^-1^]: HCM -0.5±0.2, HFpEF -0.6±0.1, Controls -1.0±0.3, p<0.01). LA booster pump function was increased in HCM while decreased in HFpEF (e_a_ [%]: HCM 11.7±4.0, HFpEF 4.5±2.9, Controls 7.8±2.5, p<0.01; SR_a_ [s^-1^]: HCM -1.2±0.4, HFpEF -0.5±0.2, Controls -0.9±0.3, p<0.01). Observer variability was excellent for all strain and SR parameters on an intra- and inter-observer level as determined by Bland-Altman, coefficient of variation and intraclass correlation coefficients.

## Conclusions

CMR-FT reliably quantifies LA longitudinal strain and SR from standard SSFP cine images. CMR-FT based atrial performance analysis discriminated between patients with impaired left ventricular relaxation and healthy controls. CMR-FT derived atrial deformation analysis seems a promising novel approach for the study of atrial performance and physiology in health and disease states.

## Funding

N/A.

